# Prediction of Hexaconazole Concentration in the Top Most Layer of Oil Palm Plantation Soil Using Exploratory Data Analysis (EDA)

**DOI:** 10.1371/journal.pone.0166203

**Published:** 2017-01-06

**Authors:** Zainol Maznah, Muhamad Halimah, Mahendran Shitan, Provash Kumar Karmokar, Sulaiman Najwa

**Affiliations:** 1 Analytical and Quality Development Unit, Product Development and Advisory Services Division (PDAS), Malaysian Palm Oil Board (MPOB), Persiaran Institusi, Bandar Baru Bangi Kajang, Selangor, Malaysia; 2 Laboratory of Computational Statistics and Operations Research, Institute for Mathematical Research, University Putra Malaysia, UPM Serdang, Selangor, Malaysia; 3 Department of Statistics, University of Rajshahi, Rajshahi, Bangladesh; National University of Ireland—Galway, IRELAND

## Abstract

*Ganoderma boninense* is a fungus that can affect oil palm trees and cause a serious disease called the basal stem root (BSR). This disease causes the death of more than 80% of oil palm trees midway through their economic life and hexaconazole is one of the particular fungicides that can control this fungus. Hexaconazole can be applied by the soil drenching method and it will be of interest to know the concentration of the residue in the soil after treatment with respect to time. Hence, a field study was conducted in order to determine the actual concentration of hexaconazole in soil. In the present paper, a new approach that can be used to predict the concentration of pesticides in the soil is proposed. The statistical analysis revealed that the Exploratory Data Analysis (EDA) techniques would be appropriate in this study. The EDA techniques were used to fit a robust resistant model and predict the concentration of the residue in the topmost layer of the soil.

## Introduction

Oil palm is the most important agricultural sector in Malaysia and it is contributing significantly to the economy of the country. In 2015, the total production of crude palm oil (CPO) recorded was 19,961,581 tons as higher compared in 2014 was 19,667,016 tons [1]. In order to produce this large volume of quality oil products, the concerned authorities such as the palm oil producers need to be careful about their production of palm oil. The application of chemicals such as fertilizers for nutrient requirements and pesticides for crop protection on oil palm is necessary in this regard. The use of pesticides is mainly for controlling weeds, pest, and fungus. Among the pesticides, cypermethrin, deltamethrin, endosulfan, fluroxypyr-MHE, chlorpyrifos, thiram, hexaconazole, etc. are notable and several studies have been carried out to investigate their behaviours [2–5].

Nowadays, the basal stem rot (BSR) which is caused by the fungus *Ganoderma boninense* is the most serious disease of oil palm in Malaysia. The cases of BSR are reported in Johor, Negeri Sembilan and Malacca in Malaysia [6–7]. The cases of BSR have also been reported in other countries like Africa, Papua New Guinea, Indonesia, and Thailand [8]. This disease causes the death of the oil palm trees in more than 80% cases midway through their economic life [9]. The primary infection of oil palms by the species of *Ganoderma* is due to the direct contact of living roots with colonized debris [10]. Among the different techniques, the best approach to control this disease consists of the removal of infected palms, soil mounding, fungicide treatment or a combination of these methods. The applications of chemical treatments are considered as the immediate short-term control measures. The use of this systemic fungicide, together with an appropriate technique of application may help to reduce the progress of the BSR [11]. Hexaconazole is one of the fungicides which can be used against the *Ganoderma* species [5,10–11] and applied by the soil drenching method. It will be of interest to know the concentration of the residue in the soil (after treatment) with respect to time.

The term Exploratory Data Analysis (EDA) was introduced by John W. Tukey who had shown how simple graphical and qualitative techniques can be used to open-mindedly explore data. The EDA can help to improve the results of statistical hypothesis testing by forcing one to look at unbiased data before formulating hypotheses which are subsequently tested using the conventional statistic (confirmatory data analysis) [12]. EDA is a specific traditional data analysis tool was introduced by John Tukey and his associates in the early 1960s while Behrens [13] discussed on the philosophical underpinning and general heuristics of it. The EDA typically begins by examining each variable individually, combing through the data, checking the shapes of distributions and looking for outliers and rogue values. Then, the exploratory data analyst turns to look at relationships between pairs of variables and finally considers multivariate relationships [14].

It has been over 37 years since the Exploratory Data Analysis (EDA) was introduced. Since that time, a number of publications integrating the EDA into the multidiscipline of science have been published [15–18]. Numerous publications discussing on the environmental behaviour of pollutant and agrochemical have been studied. The agricultural scientists routinely manage sizeable amounts of scientific data originating from the agricultural field [3–5], lab experimentation [19–20], observation [21–22], computer models [23–24], and simulations [25–26].

However, the exploitation of EDA in environmental pollution such as pesticides, polycyclic aromatic hydrocarbons (PAH) and air pollution has never been reported. The prediction of concentrations of a pesticide in the soil, surface water, and ground water often involved the use of mathematical simulation models such as VARLEACH [27–28], PESTLA [29], PELMO [30] and LEACHP [31]. The present paper reports a field study conducted in order to determine and predict the hexaconazole concentration in the real environment conditions in the top most layer of the soil. Pesticide degradation in the field can then be predicted on the basis of these parameters and actual or predicted on-site temperature and moisture data. Therefore, we decided to take different approaches based on the EDA as an alternative. We started our analysis with the data obtained from the field experiment and transformed the data to construct the statistical model.

## Methodology

Ethics Statement: N/A

### Experimental site

The experimental site, as described by our previous work [3], is located in Bangi Lama, Selangor (101°47’E, 02°54’N). The research station is owned by Universiti Kebangsaan Malaysia (UKM) and jointly developed by the Malaysian Palm Oil Board (MPOB). The trial field consists of 225 DxP palm trees of seven years old. A complete randomized block was used as the experimental design with three replicates for each plot to accommodate three treatments namely, the recommended dosage (0.639 kg ha^-1^), double the recommended dosage (1.278 kg ha^-1^) and control (without fungicide treatment). The commercial grade of hexaconazole (Anvil^®^) was applied to the experimental plots using the soil drenching method.

#### Soil sampling

The soil samples were collected from 0–50 cm (0–10, 10–20, 20–30, 30–40 and 40–50 cm) depth using a soil auger. However, in the present study only the top most layer, 0–10 cm depth of soil was used for analyses. The soil samples were collected on day 0 (on the day of spraying), 1, 3, 7, 21, 70, 90 and 120 after treatment with three replicates on the day of sampling. The soil samples were air-dried, sieved through a 2-mm mesh and stored in black polyethylene bags at -4°C prior to analysis.

#### Climate

**Fig 1:**
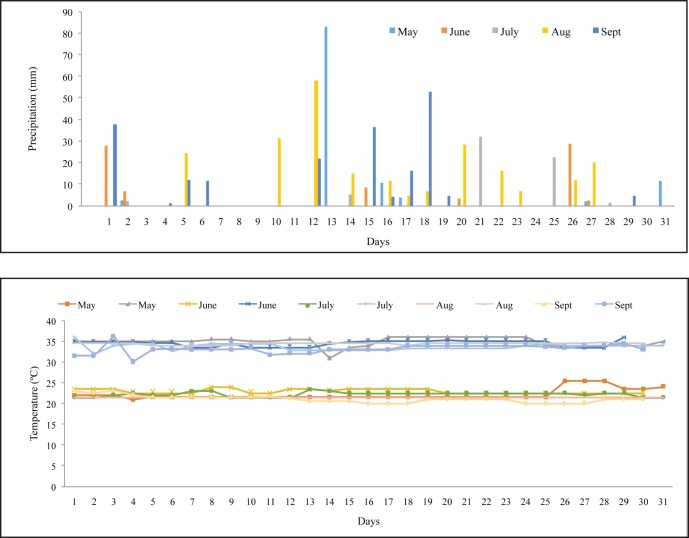
Daily precipitation and temperature

Daily rainfall and temperature were recorded from May to September 2009. The amounts of rainfall recorded were 115.1 mm, 78.90 mm, 66.20 mm, 237.70 mm and 205.5 mm for May, June, July, August, and September, respectively (Fig 1). The monthly mean of the maximum temperature values ranged from 33.8°C to 34.9°C as observed during the study period (Fig 1).

#### Recovery study

The soil sample (25 g) was treated with five concentration levels of hexaconazole viz. 0.8, 0.5, 0.2, 0.1 and 0.01 mg kg^-1^. The pesticide was then extracted from the soil to determine the hexaconazole residue for the quantification by using a gas chromatography.

#### Determination of hexaconazole in soil

The hexaconazole was determined using a similar method as in the earlier published papers [3, 5]. Twenty-five grams of soil were shaken with 100 mL of dichloromethane and placed in an ultrasonic bath for half an hour. The extract was filtered from dichloromethane using a filter paper (Whatman 4) which contains sodium sulfate. Then, 50 mL of extract solution was evaporated to make up to 10 mL. The extract was then shifted to the graduated micro-vial to gain dryness by nitrogen gas. Next, the residue was re-dissolved with 1 mL acetone before being injected into the GC-ECD. The standard hexaconazole solution was used to quantify the analyte where each solution was injected twice with five replications.

#### Gas chromatography (GC) analysis

The analysis of hexaconazole was carried out by using the gas chromatograph (GC) (HP 6890) fitted with electron capture detector (ECD) and auto-sampler injector. The capillary column used was HP 5% MS column (30 m x 0.25 mm ID, 0.25 mm film thickness). The sample volumes of 2.0 μL were injected into the programmable splitless injector. The instrumental conditions used in the present study were similar to the earlier published papers [3, 5].

#### Exploratory data analysis

For any given data set, traditional statistical methods like the Method of Least Squares can be used to obtain parameter estimates where the unknown parameters are estimated by minimizing the sum of the residual squares. However, the traditional least square estimators are based on some assumptions. If the data violates some of the assumptions, then the estimates and results can be misleading. Although the method of least squares often gives optimal estimates of the unknown parameters, it is very sensitive to the outliers and influential observations. Outliers can sometimes be a serious problem and consequently the result can mislead the prediction and validation of the fitted model. In such a case, some distributional assumptions must be made. Usually, it is assumed that the errors are normally distributed with zero mean and constant variance. The statistical inference based on the normality assumption is known to be vulnerable to outliers [32]. Secondly, it is assumed that the errors are uncorrelated and it would also be desirable to have a good number of observations, though not absolutely necessary.

Various diagnostic checking (behaviour of residuals) of the fitted models are seen in the literature with the help of which validation of a fitted model should be judged. The diagnostic plots are effective tools for checking the adequacy of data set to the fitted regression models. Residuals are assumed to be independent of the fitted values, meaning that the correlation between residuals and fitted values should be zero.

Subsequently, the effect of violation of the assumptions for the study data sets will be shown. In particular, the data sets are not normally distributed and diagnostic analysis revealed the poorly fitted models by traditional statistical methods. Furthermore, each data set comprised of eight observations only.

Hence, it is useful to search an alternative procedure where this small number of data points could be modelled properly. The alternative method suggested is a resistant fit after straightening out the plot. Details about straightening out plots and fitting resistant lines can be found in several references [33–35]. A resistant fit is a tool of EDA which does not rely too much on assumptions and is robust. First, one should check the half-slope ratio and if this ratio is not approximately 1 then a transformation is required. Usually, the Ladder of Powers is used for re-expressing the data. Once, the half-slope ratio is reasonably close to 1, and then a linear resistant fit can be done.

## Results and Discussion

### Recovery study

The recovery and relative standard deviation percent of hexaconazole spiked in soil samples at levels of 0.8, 0.5, 0.2, 0.1 and 0.01 mg kg^-1^ were 100 ± 1.92%, 105 ± 3.74%, 100 ± 5.64%, 102. ± 1.20% and 106 ± 4.28%, respectively. The detection limit of hexaconazole was 0.2 μg L^-1^.

### Field study

The data was collected from the topmost layer of the experimental plot of soil and are shown in Table 1. The soil in the trial plot was characterized by the sandy loam texture containing 27.29% clay, 62.62% sand, 10.09% silt, 0.86% total carbon and a cation exchange capacity (CEC) of 6.55.

**Table 1:** Data obtained from the field study

Concentration of hexaconazole (mg kg^-1^)	Days							
	0	1	3	7	21	70	90	120
Recommended dosage	1.578	1.850	1.283	1.508	1.189	0.981	0.549	0.310
Double recommended dosage	2.795	2.993	2.479	2.208	2.303	1.586	1.257	0.310

### Statistical modelling for the recommended dosage

**Fig 2:**
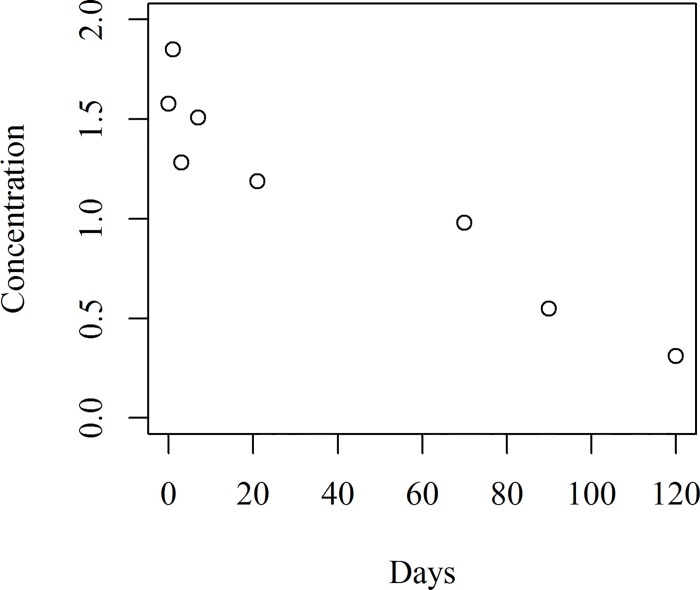
Scatter plot of hexaconazole concentration for the recommended dosage

A scatter plot of the concentration of hexaconazole (mg kg^-1^) against time is given in Fig 2. It is clear from the figure that the concentration declined with respect to time. The commonly used models namely the Linear, Exponential, Power, Logarithmic (natural and base 10) and Log-linear models have been fitted to the above data by the traditional OLS method. The results of the fitted models appear in Table 2. However, these models were not very useful since the normality assumptions used in building these models were violated, as discussed below.

**Table 2:** OLS fitted models

Name of Model	Fitted Model
Linear	*Conc*. = 1.563–0.010*Day*
Exponential	*Conc*. = 1.635*e* ^-0.012*Day*^
Power	*Conc*. = 1.654*Day* ^-0.214^
Natural Log (ln)	*Conc*. = 1.613–0.201 ln(*Day*)
Common Log (log_10_)	*Conc*. = 1.610–0.462 log_10_(*Day*)
Log-linear	*Conc*. = 0.491–0.012*Day*

**Fig 3:**
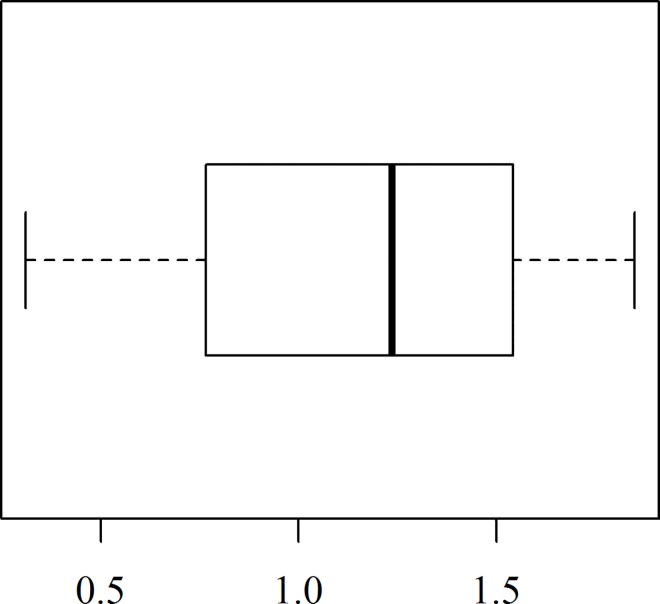
Box plot of the data set

**Fig 4:**
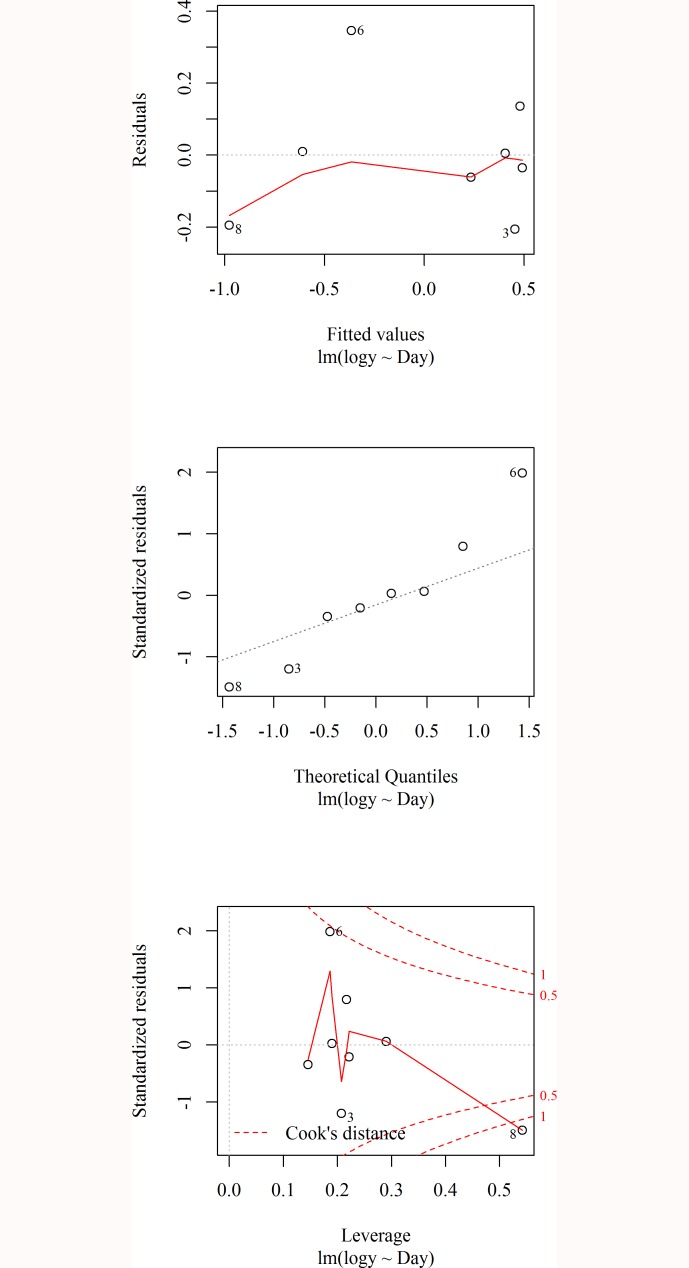
Diagnostics plot of the fitted regression model

A box plot of the data is shown in Fig 3 indicating that the data is skewed to the left. The skewness value is -0.505 and kurtosis value 2.350 also supported the result of the box plot. The skewness value for a normal distribution is zero and kurtosis is 3. Therefore, there is evidence that the data was not normally distributed. The diagnostic plots of OLS residuals are shown in Fig 4.

The residual versus fitted plot is shown in the top panel in Fig 4. The observation numbers 6 and 8 may be suspected as the potential outliers from the figure. The Q-Q Normality plot as shown in the middle panel in Fig 4 identified that the same observations were somewhat far from the straight line indicating that these points may be the source of violation of normality assumption. An observation with an extreme value of a predictor variable is known as a high leverage point and it has an unusually large effect on the estimated regression coefficients. If the model is fitted in the presence of such observations, it may mislead the whole inference. The plot of the residuals versus leverage shown in the bottom panel of Fig 2 indicates that observation 8 was outside the boundary line while observation 6 was close to the boundary line. This means that the data set had some leverage value problems, also.

Since it was shown that the data sets were not normally distributed and diagnostic analysis revealed the presence of outliers together with the leverage problems, the traditional least square regression methods may not be suitable to analyse this data set.

Hence, as an alternative, the EDA techniques named as the resistant fit were used in this study after straightening out the plot. For an appropriate resistant fitted model, the examination of the half-slope ratio is very essential. The half-slope ratio for this data set was found 0.596 which is not close to 1, indicating that the data was not linear. Hence, some sort of transformation was required for this data set. A log_*e*_ transformation for the concentration was chosen later and the half-slope ratio was recalculated and it was found to be 0.929 which is reasonably close to 1 and hence, the fitted resistant line is,

lnConc.=0.456−0.012Day (1)

In terms of the concentration the fitted model is

$$Conc.=e^{\left(0.456-0.012Day\right)}$$ (2)

**Fig 5:**
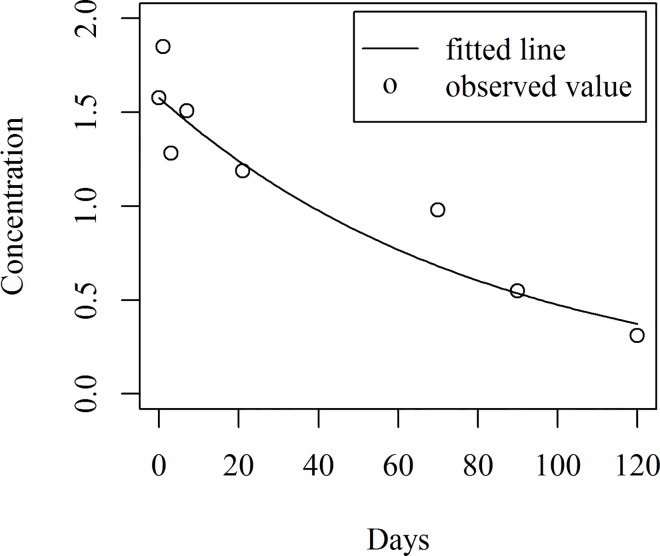
Fitted line with observed value of concentration

Fig 5 shows the fitted model together with the observed data points while the fitted values along with the residuals are shown in Table 3.

**Table 3:** Fitted values and residuals

Day	Observed values	Fitted values	Residuals
0	1.578	1.578	0.000
1	1.850	1.559	0.291
3	1.283	1.522	-0.239
7	1.508	1.451	0.057
21	1.189	1.226	-0.037
70	0.981	0.681	0.300
90	0.549	0.536	0.013
120	0.310	0.374	-0.064

The *H*-spread value for the residuals was 0.224. The procedure of computation of the *H*-spread value can be found in [33]. The predicted intervals were constructed by using the equation,

PredictedConc.±1.5H−spreadvalueofresiduals

**Fig 6:**
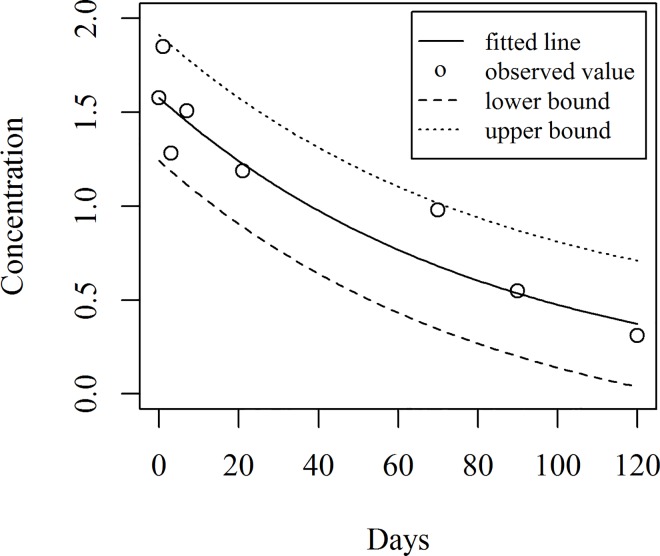
Fitted line and predicted intervals

The predicted concentration of hexaconazole and corresponding predicted intervals are given in Table 4. Fig 6 shows the fitted line and predicted intervals together with the observed data points.

**Table 4:** Predicted values and intervals for the recommended dosage

Day	Predicted	Predicted	Day	Predicted	Predicted	Day	Predicted	Predicted
	Value	Interval		Value	Interval		Value	Interval
0	1.578	(1.242, 1.914)	41	0.965	(0.629, 1.301)	82	0.590	(0.254, 0.926)
1	1.559	(1.223, 1.895)	42	0.953	(0.617, 1.289)	83	0.583	(0.247, 0.919)
2	1.540	(1.204, 1.876)	43	0.942	(0.606, 1.278)	84	0.576	(0.240, 0.912)
3	1.522	(1.186, 1.858)	44	0.931	(0.595, 1.267)	85	0.569	(0.233, 0.905)
4	1.504	(1.168, 1.840)	45	0.919	(0.583, 1.255)	86	0.562	(0.226, 0.898)
5	1.486	(1.150, 1.822)	46	0.908	(0.572, 1.244)	87	0.555	(0.219, 0.891)
6	1.468	(1.132, 1.804)	47	0.898	(0.562, 1.234)	88	0.549	(0.213, 0.885)
7	1.451	(1.115, 1.787)	48	0.887	(0.551, 1.223)	89	0.542	(0.206, 0.878)
8	1.433	(1.097, 1.769)	49	0.876	(0.540, 1.212)	90	0.536	(0.200, 0.872)
9	1.416	(1.080, 1.752)	50	0.866	(0.530,1.202)	91	0.529	(0.193, 0.865)
10	1.399	(1.063, 1.735)	51	0.856	(0.520, 1.192)	92	0.523	(0.187,0.859)
11	1.383	(1.047, 1.719)	52	0.845	(0.509, 1.181)	93	0.517	(0.181,0.853)
12	1.366	(1.030, 1.702)	53	0.835	(0.499, 1.171)	94	0.511	(0.175, 0.847)
13	1.350	(1.014, 1.686)	54	0.825	(0.489, 1.161)	95	0.505	(0.169, 0.841)
14	1.334	(0.998, 1.670)	55	0.815	(0.479, 1.151)	96	0.499	(0.163, 0.835)
15	1.318	(0.982, 1.654)	56	0.806	(0.470, 1.142)	97	0.493	(0.157, 0.829)
16	1.302	(0.966, 1.638)	57	0.796	(0.460, 1.132)	98	0.487	(0.151, 0.823)
17	1.287	(0.951, 1.623)	58	0.787	(0.451, 1.123)	99	0.481	(0.145, 0.817)
18	1.271	(0.935, 1.607)	59	0.777	(0.441, 1.113)	100	0.475	(0.139, 0.811)
19	1.256	(0.920, 1.592)	60	0.768	(0.432, 1.104)	101	0.470	(0.134, 0.806)
20	1.241	(0.905, 1.577)	61	0.759	(0.423, 1.095)	102	0.464	(0.128, 0.800)
21	1.226	(0.890, 1.562)	62	0.750	(0.414, 1.086)	103	0.458	(0.122, 0.794)
22	1.212	(0.876, 1.548)	63	0.741	(0.405, 1.077)	104	0.453	(0.117, 0.789)
23	1.197	(0.861, 1.533)	64	0.732	(0.396, 1.068)	105	0.448	(0.112, 0.784)
24	1.183	(0.847, 1.519)	65	0.723	(0.387, 1.059)	106	0.442	(0.016, 0.778)
25	1.169	(0.833, 1.505)	66	0.715	(0.379, 1.051)	107	0.437	(0.101, 0.773)
26	1.155	(0.819, 1.491)	67	0.706	(0.370, 1.042)	108	0.432	(0.096, 0.768)
27	1.141	(0.805, 1.477)	68	0.698	(0.362, 1.034)	109	0.427	(0.091, 0.763)
28	1.127	(0.791, 1.463)	69	0.689	(0.353, 1.025)	110	0.421	(0.085, 0.757)
29	1.114	(0.778, 1.450)	70	0.681	(0.345, 1.017)	111	0.416	(0.080, 0.752)
30	1.101	(0.765, 1.437)	71	0.673	(0.337, 1.009)	112	0.411	(0.075, 0.747)
31	1.088	(0.752, 1.424)	72	0.665	(0.329, 1.001)	113	0.407	(0.071, 0.743)
32	1.075	(0.739, 1.411)	73	0.657	(0.321, 0.993)	114	0.402	(0.066, 0.738)
33	1.062	(0.726, 1.398)	74	0.649	(0.313, 0.985)	115	0.397	(0.061, 0.733)
34	1.049	(0.713, 1.385)	75	0.641	(0.305, 0.977)	116	0.392	(0.056, 0.728)
35	1.037	(0.701, 1.373)	76	0.634	(0.298, 0.970)	117	0.388	(0.052, 0.724)
36	1.024	(0.688, 1.360)	77	0.626	(0.290, 0.962)	118	0.383	(0.047, 0.719)
37	1.102	(0.676, 1.348)	78	0.619	(0.283, 0.955)	119	0.378	(0.042, 0.714)
38	1.000	(0.664, 1.336)	79	0.611	(0.275, 0.947)	120	0.374	(0.038, 0.710)
39	0.988	(0.652, 1.324)	80	0.604	(0.268, 0.940)			
40	0.976	(0.640, 1.312)	81	0.597	(0.261, 0.933)			

### Statistical Modelling for the double recommended dosage

**Fig 7:**
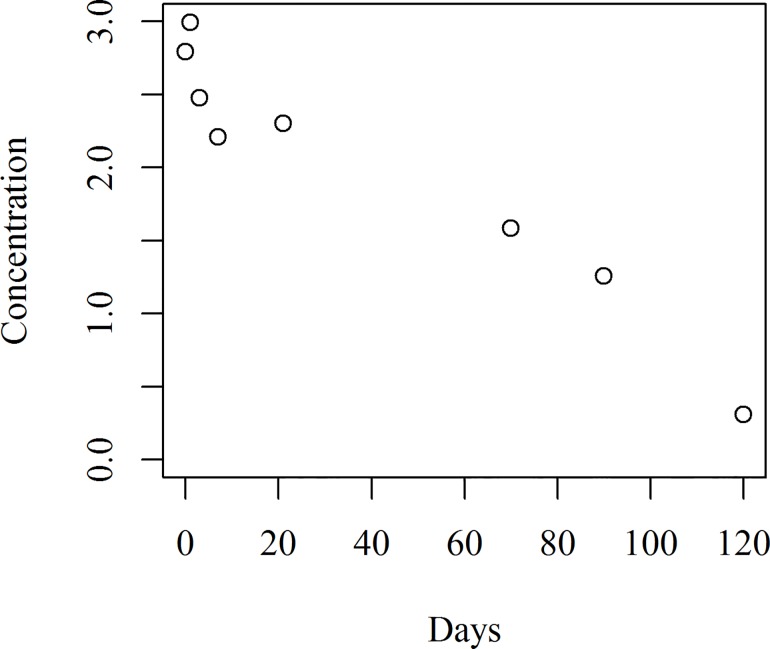
Scatter plot of hexaconazole concentration for the double recommended dosage

The scatter plot of the hexaconazole concentration of the double dosage is given in Fig 7. The half-slope ratio (*Conc*. against *Day*) found was 0.317 which is not close to 1 indicating that the data was not linear. Hence, some sort of transformation was required and a transformation was chosen for the *Day* raised to the power of 0.4. The half-slope ratio of the transformed line found was 1.007 which is very close to 1.

Thus, the fitted resistant line for double dosage is

$$Conc.=2.901-0.272{\left(Day\right)}^{0.4}$$ (3)

**Fig 8:**
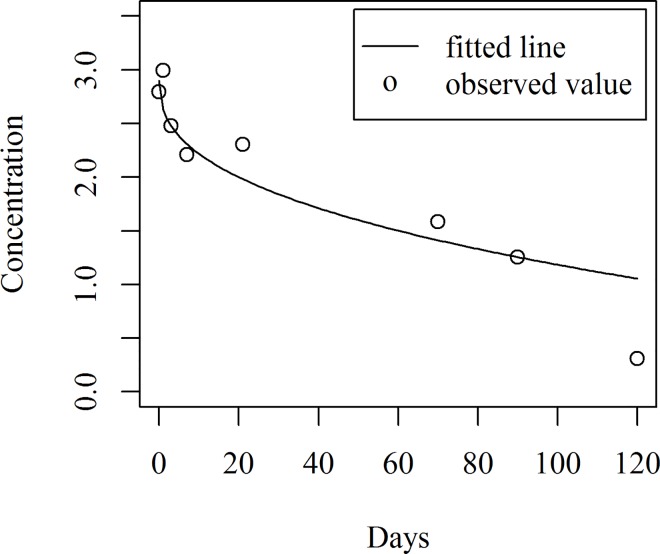
Fitted line with observed value of concentration

Fig 8 shows the fitted model together with the observed data points. The fitted values along with the residuals of the fitted models for the double dosage of concentration are shown in Table 5.

**Table 5:** Fitted values and residuals

Day	Observed values	Fitted values	Residuals
0	2.795	2.901	-0.106
1	2.993	2.629	0.364
3	2.479	2.479	0.000
7	2.208	2.309	-0.101
21	2.303	1.982	0.321
70	1.586	1.413	0.173
90	1.257	1.256	0.001
120	0.310	1.055	-0.745

Here the *H*-spread value for the residuals was 0.350. Again, the predicted intervals were constructed by using the equation,

PredictedConc.±1.5H−spreadvalueofresiduals

**Fig 9:**
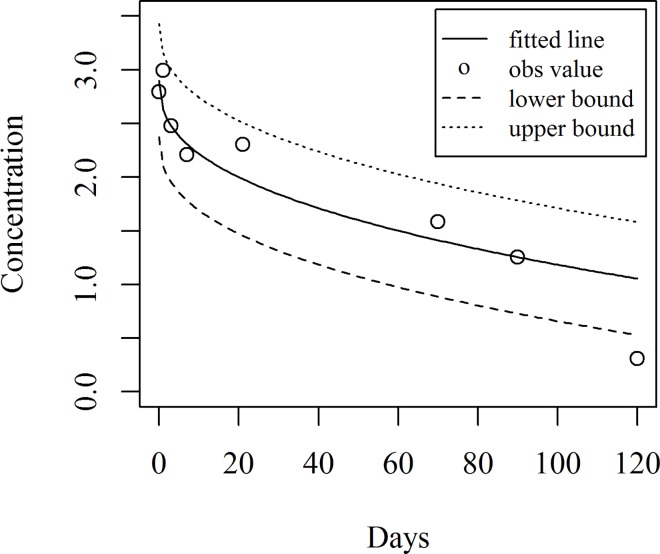
Fitted line and predicted intervals

**Fig 10:**
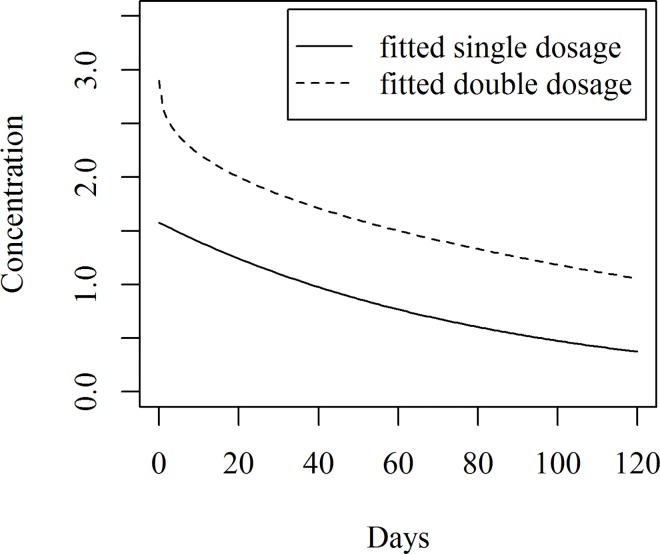
Comparison of fitted lines

The predicted concentration and corresponding predicted intervals are given in Table 6 and the fitted line and predicted intervals together with the observed data points are shown in Fig 9. To measure the decline rate of hexaconazole concentration for both single and double dosages, both the models were plotted together as shown in Fig 10. It can be seen that the rate of decline was not the same, based on these models. The double dosage declines at a faster rate than the single dosage during the first week after treatment.

**Table 6:** Predicted values and intervals for the double recommended dosage

Day	Predicted	Predicted	Day	Predicted	Predicted	Day	Predicted	Predicted
	Value	Interval		Value	Interval		Value	Interval
0	2.901	(2.374, 3.427)	41	1.700	(1.173, 2.226)	82	1.316	(0.789, 1.842)
1	2.629	(2.102, 3.155)	42	1.688	(1.161, 2.214)	83	1.308	(0.782, 1.835)
2	2.542	(2.016, 3.069)	43	1.677	(1.150, 2.203)	84	1.300	(0.774, 1.827)
3	2.479	(1.952, 3.005)	44	1.665	(1.139, 2.192)	85	1.293	(0.766, 1.819)
4	2.427	(1.901, 2.954)	45	1.654	(1.128, 2.181)	86	1.285	(0.759, 1.812)
5	2.383	(1.857, 2.910)	46	1.643	(1.117, 2.170)	87	1.278	(0.751, 1.804)
6	2.344	(1.818, 2.871)	47	1.632	(1.106, 2.159)	88	1.270	(0.744, 1.797)
7	2.309	(1.782, 2.835)	48	1.621	(1.095, 2.148)	89	1.263	(0.736, 1.789)
8	2.276	(1.750, 2.803)	49	1.611	(1.804, 2.137)	90	1.256	(0.729, 1.782)
9	2.246	(1.719, 2.772)	50	1.600	(1.074, 2.127)	91	1.248	(0.722, 1.775)
10	2.218	(1.691, 2.744)	51	1.590	(1.064, 2.117)	92	1.241	(0.715, 1.768)
11	2.191	(1.665, 2.718)	52	1.580	(1.053, 2.106)	93	1.234	(0.707, 1.760)
12	2.166	(1.640, 2.693)	53	1.570	(1.043, 2.096)	94	1.227	(0.700, 1.753)
13	2.142	(1.616, 2.669)	54	1.560	(1.033, 2.086)	95	1.220	(0.693, 1.746)
14	2.119	(1.593, 2.646)	55	1.550	(1.023, 2.076)	96	1.213	(0.686, 1.739)
15	2.097	(1.571, 2.624)	56	1.540	(1.014, 2.067)	97	1.206	(0.679, 1.732)
16	2.076	(1.550, 2.603)	57	1.530	(1.004, 2.057)	98	1.199	(0.672, 1.725)
17	2.056	(1.530, 2.583)	58	1.521	(0.994, 2.047)	99	1.192	(0.665, 1.718)
18	2.037	(1.510, 2.563)	59	1.511	(0.985, 2.038)	100	1.185	(0.658, 1.711)
19	2.018	(1.491, 2.544)	60	1.502	(0.975, 2.028)	101	1.178	(0.651, 1.704)
20	1.999	(1.473, 2.526)	61	1.493	(0.966, 2.019)	102	1.171	(0.645, 1.698)
21	1.982	(1.455, 2.508)	62	1.483	(0.957, 2.010)	103	1.164	(0.638, 1.691)
22	1.964	(1.438, 2.491)	63	1.474	(0.948, 2.001)	104	1.158	(0.631, 1.684)
23	1.948	(1.421, 2.474)	64	1.465	(0.939, 1.992)	105	1.151	(0.624, 1.677)
24	1.931	(1.405, 2.458)	65	1.456	(0.930, 1.983)	106	1.144	(0.618, 1.671)
25	1.915	(1.389, 2.442)	66	1.448	(0.921, 1.974)	107	1.138	(0.611, 1.664)
26	1.900	(1.373, 2.426)	67	1.439	(0.912, 1.965)	108	1.131	(0.605, 1.658)
27	1.884	(1.358, 2.411)	68	1.430	(0.904, 1.957)	109	1.125	(0.598, 1.651)
28	1.870	(1.343, 2.396)	69	1.422	(0.895,1.948)	110	1.118	(0.592, 1.645)
29	1.855	(1.329, 2.382)	70	1.413	(0.886, 1.939)	111	1.112	(0.585, 1.638)
30	1.841	(1.314, 2.367)	71	1.405	(0.878, 1.931)	112	1.105	(0.579, 1.632)
31	1.827	(1.300, 2.353)	72	1.396	(0.870, 1.923)	113	1.099	(0.572, 1.625)
32	1.813	(1.286, 2.339)	73	1.388	(0.861, 1.914)	114	1.092	(0.566, 1.619)
33	1.800	(1.273, 2.326)	74	1.380	(0.853, 1.906)	115	1.086	(0.560, 1.613)
34	1.786	(1.260, 2.313)	75	1.371	(0.845, 1.898)	116	1.080	(0.553, 1.606)
35	1.773	(1.247, 2.300)	76	1.363	(0.837, 1.890)	117	1.074	(0.547, 1.600)
36	1.761	(1.234, 2.287)	77	1.355	(0.829, 1.882)	118	1.067	(0.541, 1.594)
37	1.748	(1.221, 2.274)	78	1.347	(0.821, 1.874)	119	1.061	(0.535, 1.588)
38	1.736	(1.209, 2.262)	79	1.339	(0.813, 1.866)	120	1.055	(0.528, 1.581)
39	1.723	(1.197, 2.250)	80	1.331	(0.805, 1.858)			
40	1.711	(1.185, 2.238)	81	1.324	(0.797, 1.850)			

## Conclusion

Statistical models were constructed for hexaconazole concentration for the topmost layer of the soil, both for the single and double dosages. Transformations and fitted resistant lines were applied for these dosages. The usefulness of these models are that they are simple, robust and do not rely on too many assumptions. The predicted values and intervals for both single and double dosages were also constructed and it was found that EDA can serve as a guide in predicting the concentration of hexaconazole after treatment. The advantages of using these prediction models are that it will save the time and effort in collecting field samples, cost of manpower and chemicals and indicate sampling interval after treatment. As these models were developed with only eight observations, it can be improved gradually if more observations are available. The inclusion of some other important variables like rainfall, preferential flow, microorganisms, etc. can also be considered in the future.
